# Curse of rarity for autonomous vehicles

**DOI:** 10.1038/s41467-024-49194-0

**Published:** 2024-06-05

**Authors:** Henry X. Liu, Shuo Feng

**Affiliations:** 1 Department of Civil and Environmental Engineering, University of Michigan, Ann Arbor, MI USA; 2 Mcity, University of Michigan, Ann Arbor, MI USA; 3 Department of Automation, Beijing National Research Center for Information Science and Technology, Tsinghua University, Beijing, China

**Keywords:** Civil engineering, Mechanical engineering

## Abstract

The curse of rarity—the rarity of safety-critical events in high-dimensional variable spaces—presents significant challenges in ensuring the safety of autonomous vehicles using deep learning. Looking at it from distinct perspectives, the authors identify three potential approaches for addressing the issue.

## Teaser

The curse of rarity—the rarity of safety-critical events in high-dimensional variable spaces—presents significant challenges in ensuring the safety of autonomous vehicles using deep learning. Looking at it from distinct perspectives, we identify three potential approaches for addressing the issue.

The concept of autonomous vehicles (AVs) has been around for about a century. Over the past two decades, AVs have attracted extensive attention from academic institutions, government agencies, professional organizations, and industries. By 2015, multiple companies had announced that they would mass-produce AVs before 2020 (Ref. ^1^). However, the reality has not lived up to expectations, and there are currently no commercially available SAE Level 4 (Ref. ^2^) AVs. One of the main reasons is the significant gap in safety performance of AVs^1^. This gap poses a major challenge as AVs struggle to effectively handle a multitude of rare safety-critical events, despite the accumulation of millions of testing miles on public roads. The occurrence of these events, characterized by a probability distribution resembling a long tail that is far from the head or central part of the distribution, is commonly referred to as the long-tail challenge for AV safety^3,4^. The catchphrase “long-tail challenge” for AV safety, however, is frequently used in a handwaving manner without a formal definition in the literature. This lack of understanding impedes progress in resolving the issue.

In this Comment, we uncover that the shape of the probability distribution for safety-critical occurrences, whether it exhibits a long tail or not, is not essential to the issue at hand. Instead, the primary challenge in defining the problem stems from the rareness of safety-critical situations in highly complex driving environments, which encompass various factors such as different weather conditions, diverse road infrastructures, and behavioral distinctions among road users. The safety-critical circumstances may arise due to a variety of reasons, such as misidentification of an unknown object or inaccurate prediction of nearby pedestrian’s movement, all of which have a low probability of occurrence. We term this challenge as the curse of rarity (CoR) and mathematically define CoR for a generic deep learning problem, which is commonly used for perception, behavior modelling, prediction and decision making in AVs. CoR emerges from the combination of rare occurrence of safety-critical situations and the vast number of variables involved, resulting in a compounding effect. Such an effect hinders the ability of deep learning models to perform safely in real-time^5,6^.

In the following, we elaborate the CoR in different AV tasks including perception, prediction, planning, as well as validation and verification. Based on these analyses, we discuss potential solutions towards addressing the CoR. We hope that this Comment can provide a better understanding of the safety challenges faced by the AV community, and a rigorous formulation of CoR can help accelerate the development and deployment of AVs as well as other safety-critical autonomous systems.

## What is the curse of rarity?

The basic concept of CoR is that the occurrence probability for the events of interest in high-dimensional space is so rare that most available data contain very little information of the rare events. Therefore, it is hard for a deep-learning model to learn, since valuable information of rare events could be buried under a large amount of normal data. It becomes particularly challenging to improve safety performance because better safety performance also means a lower frequency of safety-critical events, which makes it more difficult for the deep-learning model to learn. An illustration of CoR can be found in Box 1.

Box 1 Curse of rarity for deep learning models

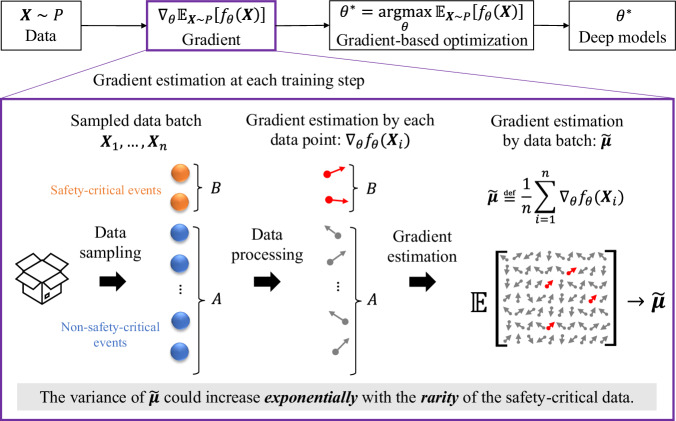

The key to deep learning is to obtain the optimal parameters θ^*^ of neural networks by optimizing the expectations of the objective function *f*_*θ*_(∙) over the data *X* with an underlying distribution *P*. To solve this optimization problem, the most commonly used approach is based on the gradient descent (see Chapter 8 in ref. ^19^). Existing approaches estimate the gradient with Monte Carlo estimation^20^ using a batch of data at each training step. However, the estimation variance could increase exponentially with the rarity of safety-critical events (see Theorem 1 in Methods), resulting in the curse of rarity. This analysis is applicable to various deep-learning approaches. In the case of deep reinforcement learning, the data consists of the state-action pairs, and the objective function is based on the reward function (see our previous work^5^ for details); whereas in deep supervised learning, the data comprises the labelled data, and the objective function is based on the loss function. More details can be found in the Methods section.

## What challenges does it bring for autonomous vehicles?

In this section, we elaborate on the CoR in various aspects of AVs including perception, prediction, planning, validation and verification.

### Perception

Deep learning methods have been extensively utilized in perception tasks to acquire information and extract pertinent knowledge from the surrounding environment. The problem of imbalanced data has been studied in perception tasks, where a small portion of object classes have a large number of samples, while the remaining classes have only a few samples^3,7^. However, this issue becomes particularly challenging for safety-critical perception tasks of AVs, as the imbalance ratios are much more severe, often exceeding 10^6^ (Ref. ^8^). Existing approaches such as class rebalancing, information augmentation, and module improvement are inadequate in addressing this problem, as they can only handle a limited imbalance ratio, usually smaller than 10^3^ (Ref. ^7^). This significant difference in magnitude fundamentally transforms the problem from an imbalanced data issue to the CoR problem. Moreover, the cumulative effects of a series of perception errors could be dangerous, even if each individual error appears insignificant. For example, an object misclassification in a single frame might be less of an issue, while multiple object misclassifications in a sequence of frames may lead to safety-critical outcomes. Since the occurrence probability of such a sequence is much lower than that of any individual error, the issue of CoR becomes even more severe.

### Behavior prediction and simulation

AV’s high safety performance requirements necessitate precise behavior modeling and accurate prediction of surrounding road users. Even a minor error in predicting the behaviors of surrounding road users can be deemed unacceptable in safety-critical situations. For example, in a jaywalking scenario, precise prediction of pedestrian trajectories is crucial for AVs to avoid collisions. A small prediction error could result in either a false alarm or a missed alarm, leading to overly cautious driving decisions or overly confident decision that cause an accident. The same holds true for driving behavior simulation. Inaccuracies in simulations can lead to underestimation or overestimation of AV’s safety performance, thereby misleading the development process^9^. To achieve the required level of safety, behavior prediction models must effectively handle rare events in high-dimensional driving environments, which are prone to the CoR.

### Decision making

Deep learning techniques, such as deep imitation learning and deep reinforcement learning, have been applied in the decision-making process of AVs. However, when it comes to safety-critical scenarios, deep learning models suffers from the CoR due to the scarcity of real-world data. This scarcity may lead to severe variance in the estimation of policy gradients, thereby impeding the effectiveness of deep learning^5^. Another approach aiming to ensure the safety of decision-making involves using formal methods based on a set of assumptions. Typical assumptions include the availability of a system model, which may be characterized by bounded unknown dynamics and noise^10^. Due to the CoR, it is difficult to verify these assumptions to account for all rare safety-critical events in high-dimensional driving environments.

### Verification and validation

Verification and validation of safety performance play a crucial role in assessing the readiness of AVs for widespread deployment^5^. Prevailing approaches usually test AVs in the naturalistic driving environment through a combination of software simulation, closed test track, and on-road testing. Due to the CoR, however, hundreds of millions of miles would be required to evaluate the safety performance of AVs, which is impractical and inefficient^8^. To accelerate the process, various approaches have been developed, such as scenario-based approaches, which focus on testing AVs in purposely generated scenarios. Unfortunately, the complexity of generating spatiotemporally intricate safety-critical scenarios poses significant challenge due to the CoR. For example, it has been found that the importance-sampling-based approaches could suffer from a severe inefficiency owing to the dramatic variance for generating complex safety-critical scenarios^6^. As a result, many existing approaches are limited to handling short scenario segments with limited dynamic objects, failing to capture the full complexity and variability of real-world safety-critical events^6^.

## What are the potential solutions?

Based on the analyses and discussions above, we identify three potential approaches for solving the CoR problem, each addressing it from a distinct perspective. It is important to note that these approaches are not mutually exclusive, and combining these approaches holds immense potential in resolving the CoR issue and expediting the widespread deployment of AVs.

### Approach #1: Effective training with more rare event data

The first approach focuses on data and aims to continually improve the handling of rare events by making better use of additional data. One potential method is to utilize exclusively the data associated with rare events, which could significantly reduce the estimation variance, as stated in Theorem 1 in Methods section. However, defining and identifying rare events are challenging, as they depend on problem-specific objective functions and suffer from the spatiotemporal complexity of safety-critical autonomous systems. More importantly, theoretical foundations that can guide the utilization of rare event data remain lacking. For AV safety validation tasks, tackling the CoR issue has been attempted by developing the dense deep reinforcement learning (D2RL) approach in our prior work^5^. Theoretical and experimental results show that D2RL can dramatically reduce the variance of the policy gradient estimation, a significant step towards addressing the CoR. Another crucial concern is how to gather or generate more rare event data. Tesla proposed the concept of shadow mode testing^11^, where rare events of interest are identified by comparing human driving behavior with autonomous driving behavior, but no details are given in the literature. Other than collecting data from naturalistic driving environment, various data augmentation methods have been developed to generate safety-critical scenarios^12^.

### Approach #2: Improving capabilities of generalization and reasoning

The second approach centers around improving the generalization and reasoning capabilities of machine learning models to overcome the data insufficiency. Intuitively, as humans can learn to drive with limited experience (typically less than one hundred hours of training), future AI agents for AVs may be able to overcome the CoR without relying on extensive task-specific data. This requires an AI agent to possess both bottom-up reasoning (sensing data-driven) and top-down reasoning (cognition expectation-driven) capabilities^13^, bridging the information gap not found in the data. These requirements are in line with the development of artificial general intelligence (AGI). Recently, foundational models such as large language models (LLMs) and vision-language models (VLMs) have exhibited remarkable generalization and reasoning abilities in terms of natural language processing and visual comprehension and reasoning by employing techniques such as fully supervised fine-tuning, in-context learning, and chain of thought. By leveraging the extensive data available, LLMs and VLMs present a promising solution for enabling top-down reasoning to address the CoR issue^14^, although issues like hallucinations still need further investigations^15^.

### Approach #3: Reducing the occurrence of safety-critical events

The third approach aims to mitigate the consequences of CoR on AV systems by reducing the occurrences of safety-critical events. Potentially, one can combine traditional model-based approaches with deep learning approaches, taking advantages of the strengths of both^16^. For example, formal methods have been developed to prevent unsafe behaviors of AVs based on abstract models, potentially leading to defensive driving strategies. However, as discussed in ref. ^10,17^, multiple challenges need to be addressed to fully harness the potential of formal methods. Another approach is to enhance situational awareness by utilizing infrastructure-based sensors or cooperative awareness, aiding AVs in overcoming the limitations of their own onboard sensors. Nevertheless, effectively utilizing this additional information to achieve improved performance remains a challenging task, especially in safety-critical scenarios. Many existing approaches may even result in inferior perception and decision-making outcomes in such scenarios, due to the increased complexity and latency associated with gathering and integrating this extra information^18^.

## Methods

Let us consider a general deep learning problem that can be formulated as an optimization problem:

1 $${\max }_{\theta }{{\mathbb{E}}}_{P}\left[{f}_{\theta }\left({{{{{\boldsymbol{X}}}}}}\right)\right],$$

where $\theta \in {{\mathbb{R}}}^{d}$ denotes the parameters of a neural network, *d* is the dimension of the parameters, ${{{{{\boldsymbol{X}}}}}}\in \Omega$ denotes the training data with an underlying distribution *P*, and ${f}_{\theta }\left({{{{{\boldsymbol{X}}}}}}\right)$ denotes the objective function given the neural network *θ* and training data ***X***. To optimize the objective function, the key is to estimate the gradient of the neural network parameters at each training iteration (see Chapter 8 in ref. ^19^) as

2 $${{{{{\boldsymbol{\mu }}}}}}\mathop=^{\scriptscriptstyle\rm def}{\nabla }_{\theta }{{\mathbb{E}}}_{P}\left[{f}_{\theta }\left({{{{{\boldsymbol{X}}}}}}\right)\right]\approx \widetilde{{{{{{\boldsymbol{\mu }}}}}}} \mathop=^{\scriptscriptstyle\rm def} \frac{1}{n}{\sum }_{i=1}^{n}{\nabla }_{\theta }{f}_{\theta }\left({{{{{{\boldsymbol{X}}}}}}}_{i}\right),{{{{{{\boldsymbol{X}}}}}}}_{i}\sim P$$

where *n* denotes the number of training data samples at each iteration, ∇_*θ*_ denotes the gradient of parameters, and the approximation is obtained using the Monte Carlo method^20^. Let ${\widetilde{\mu }}^{(k)}$ denote the *k*th component of $\widetilde{{{{{{\boldsymbol{\mu }}}}}}}$, where *k* = 1,…,*d*. According to the Monte Carlo method, $\widetilde{{{{{{\boldsymbol{\mu }}}}}}}$ is an unbiased estimation of ***μ***, that is, ${{\mathbb{E}}}_{P}\left(\widetilde{{{{{{\boldsymbol{\mu }}}}}}}\right)={{{{{\boldsymbol{\mu }}}}}}$. The variance of ${\widetilde{\mu }}^{(k)}$ can be denoted as ${\sigma }_{P}^{2}({\widetilde{\mu }}^{(k)})$. To simplify the notations, we denote ${{{{{\boldsymbol{Y}}}}}} {=}^{\scriptscriptstyle\rm def} {\nabla }_{\theta }{f}_{\theta }\left({{{{{\boldsymbol{X}}}}}}\right)$ as a random vector where ${{{{{\boldsymbol{Y}}}}}}{{{{{\boldsymbol{=}}}}}}\left[{Y}_{1},\ldots,{Y}_{d}\right]\in {{\mathbb{R}}}^{d}$, so $\widetilde{{{{{{\boldsymbol{\mu }}}}}}}$ in Eq. (2) can be represented as

3 $$\widetilde{{{{{{\boldsymbol{\mu }}}}}}}=\frac{1}{n}{\sum }_{i=1}^{n}{{{{{{\boldsymbol{Y}}}}}}}_{i}.$$

Now let’s focus on a special set of deep learning problems where only a very small portion of training data (safety-critical data) can contribute effectively to the gradient estimation, while a vast majority of training data (non-safety-critical data) contributes little. To be more specific, we can define normal events *A*⊂Ω and critical but rare events B⊂Ω, where *A***∩***B*=∅ and *A*∪*B*=Ω. We can also define the corresponding indicator function ${{\mathbb{I}}}_{A}({{{{{\boldsymbol{X}}}}}})$, where ${{\mathbb{I}}}_{A}\left({{{{{\boldsymbol{X}}}}}}\right)=1$ if ***X*** belongs to the set *A* and otherwise ${{\mathbb{I}}}_{A}\left({{{{{\boldsymbol{X}}}}}}\right)=0$. ${{\mathbb{I}}}_{B}({{{{{\boldsymbol{X}}}}}})$ can be defined similarly. Then, we can obtain a new estimator of the gradient that only utilizes the samples associated with the events B as

4 $$\hat{{{{{{\boldsymbol{\mu }}}}}}}\mathop=^{\scriptscriptstyle\rm def}\frac{1}{n}{\sum }_{i=1}^{n}\left({{{{{{\boldsymbol{Y}}}}}}}_{i}\cdot {{\mathbb{I}}}_{B}\left({{{{{{\boldsymbol{X}}}}}}}_{i}\right)\right),$$

where ${\hat{\mu }}^{(k)}$ denotes the *k*th component of $\hat{{{{{{\boldsymbol{\mu }}}}}}}$ and the variance of ${\hat{\mu }}^{(k)}$ can be denoted as ${\sigma }_{P}^{2}({\hat{\mu }}^{(k)})$.

Then we have the following theorem, and the proof can be found at the end of Methods.

**Theorem 1**:

If the set *A* satisfies the following condition:

5 $${{\mathbb{E}}}_{P}\left[{{{{{\boldsymbol{Y}}}}}}\cdot {{\mathbb{I}}}_{A}\left({{{{{\boldsymbol{X}}}}}}\right)\right]={{{{{\bf{0}}}}}},$$

we have the following properties:

1. $${{\mathbb{E}}}_{P}\left(\widetilde{{{{{{\boldsymbol{\mu }}}}}}}\right)={{\mathbb{E}}}_{P}\left(\hat{{{{{{\boldsymbol{\mu }}}}}}}\right)={{{{{\boldsymbol{\mu }}}}}};$$
2. $${\sigma }_{P}^{2}({\widetilde{\mu }}^{(k)})\ge \,{\sigma }_{P}^{2}({\hat{\mu }}^{(k)})$$; and
3. ${\sigma }_{P}^{2}({\widetilde{\mu }}^{\left(k\right)})\ge {10}^{r}\cdot {\sigma }_{P}^{2}({\hat{\mu }}^{(k)})$, with the assumption

6 $${{\mathbb{E}}}_{P}\left({Y}_{k}^{2}\cdot {{\mathbb{I}}}_{B}({{{{{\boldsymbol{X}}}}}})\right)={{\mathbb{E}}}_{P}\left({Y}_{k}^{2}\right)\cdot {{\mathbb{E}}}_{P}\left({{\mathbb{I}}}_{B}\left({{{{{\boldsymbol{X}}}}}}\right)\right),\,k=1,\ldots,d,$$

where ${{{{{\rm{r}}}}}} {=}^{{{\rm{def}}}}-{\log }_{10}\left[{{{{{{\rm{E}}}}}}}_{{{{{{\rm{P}}}}}}}\left({{\mathbb{I}}}_{B}\left({{{{{\rm{X}}}}}}\right)\right)\right]$ is defined as the rarity of the events *B* in all samples with the sampling distribution *P*.

Remark 1. The condition in Eq. (5) indicates that the non-safety-critical data $({{\mathbb{I}}}_{A}\left({{{{{\boldsymbol{X}}}}}}\right)=1)$ contributes little to the gradient. Taking the AV safety testing task as an example (see ref. ^5^ for details), the key is to learn a deep model to control background vehicles to conduct adversarial maneuvers. In this case, the non-safety-critical data that could be identified by safety metrics usually contains no information for learning such adversarial maneuvers, so the condition could be satisfied. We note that the condition is primarily for the theoretical analysis to be clean and is not strictly required in practice. For example, if ${{\mathbb{E}}}_{P}\left[{{{{{\boldsymbol{Y}}}}}}\cdot {{\mathbb{I}}}_{A}\left({{{{{\boldsymbol{X}}}}}}\right)\right]$ is a near-zero value and dramatically smaller than ${{\mathbb{E}}}_{P}\left[{{{{{\boldsymbol{Y}}}}}}\cdot {{\mathbb{I}}}_{B}\left({{{{{\boldsymbol{X}}}}}}\right)\right]$, we can still find that the variance of $\widetilde{{{{{{\boldsymbol{\mu }}}}}}}$ increases dramatically with the rarity of safety-critical events.

Remark 2. Defining and identifying the events *A* and *B* are non-trivial and dependent on specific deep learning tasks. An important aspect of these definitions is the approximate fulfillment of the condition stated in Eq. (5), as explained in Remark 1. To illustrate, in the context of AV safety testing, we have chosen safety-critical states as events *B* and non-safety-critical states as events *A* (see ref. ^5^ for details). The definitions will vary across different AV tasks, warranting further exploration.

Remark 3. The assumption in Eq. (6) can be satisfied if all ${Y}_{k}^{2},k=1,\ldots,d$ are independent of the events *B*. For deep learning approaches, the gradient ${{{{{\boldsymbol{Y}}}}}}\stackrel{\scriptscriptstyle{{{{\mathrm{def}}}}}}{=}{\nabla }_{\theta }{f}_{\theta }\left({{{{{\boldsymbol{X}}}}}}\right)$ is mainly determined by the parameters *θ* of neural networks. As the parameters are usually randomly initiated, ***Y*** could have an uncertainty that is approximately independent of the events *A* and *B*, particularly at the beginning of the learning process. Therefore, the assumption could be approximately satisfied particularly at the beginning of the learning process, so the CoR hinders the effectiveness of learning from the very beginning. Again, we note that the assumption is primarily for the theoretical analysis to be clean and is not strictly required in practice.

Remark 4. The third property suggests that the variance ${\sigma }_{P}^{2}({\widetilde{\mu }}^{\left(k\right)})$ will grow exponentially with the rarity of the events *B*, provided that ${\sigma }_{P}^{2}({\hat{\mu }}^{(k)})$ does not decrease exponentially with the rarity. As the estimator $\hat{{{{{{\boldsymbol{\mu }}}}}}}$ is primarily focused on estimating the gradient using safety-critical events, its variance ${\sigma }_{P}^{2}({\hat{\mu }}^{(k)})$ will not be affected significantly by the rarity.

*Proof of Theorem 1*.

1. Proof of $${{\mathbb{E}}}_{{{{{{\boldsymbol{P}}}}}}}\left(\widetilde{{{{{{\boldsymbol{\mu }}}}}}}\right){{{{{\boldsymbol{=}}}}}}{{\mathbb{E}}}_{{{{{{\boldsymbol{P}}}}}}}\left(\hat{{{{{{\boldsymbol{\mu }}}}}}}\right){{{{{\boldsymbol{=}}}}}}{{{{{\boldsymbol{\mu }}}}}}$$**:**
  $${{\mathbb{E}}}_{P}\left(\hat{{{{{{\boldsymbol{\mu }}}}}}}\right)={{\mathbb{E}}}_{P}\left(\frac{1}{n}{\sum }_{i=1}^{n}\left({{{{{\boldsymbol{Y}}}}}}{{{{{\boldsymbol{(}}}}}}{{{{{{\boldsymbol{X}}}}}}}_{{{{{{\boldsymbol{i}}}}}}}{{{{{\boldsymbol{)}}}}}}\cdot {{\mathbb{I}}}_{B}\left({{{{{{\boldsymbol{X}}}}}}}_{i}\right)\right)\right)={{\mathbb{E}}}_{P}\left({{{{{\boldsymbol{Y}}}}}}{{{{{\boldsymbol{(}}}}}}{{{{{{\boldsymbol{X}}}}}}}_{{{{{{\boldsymbol{i}}}}}}}{{{{{\boldsymbol{)}}}}}}\cdot {{\mathbb{I}}}_{B}\left({{{{{{\boldsymbol{X}}}}}}}_{i}\right)\right)={{{{{\boldsymbol{\mu }}}}}}={{\mathbb{E}}}_{P}\left(\widetilde{{{{{{\boldsymbol{\mu }}}}}}}\right).$$
  *End of proof*.
2. Proof of $${\sigma }_{P}^{2}({\widetilde{\mu }}^{(k)})\ge \,{\sigma }_{P}^{2}({\hat{\mu }}^{(k)})$$**:**
  $${\sigma }_{P}^{2}({\hat{\mu }}^{(k)})\,={Va}{r}_{P}\left[{{{{{{\rm{Y}}}}}}}_{{{{{{\rm{k}}}}}}}\cdot {{\mathbb{I}}}_{B}\left({{{{{\boldsymbol{X}}}}}}\right)\right]={{\mathbb{E}}}_{P}\left[{{{{{{\rm{Y}}}}}}}_{{{{{{\rm{k}}}}}}}^{2}\cdot {{\mathbb{I}}}_{B}\left({{{{{\boldsymbol{X}}}}}}\right)\right]-{{\mathbb{E}}}_{P}^{2}\left[{{{{{{\rm{Y}}}}}}}_{{{{{{\rm{k}}}}}}}\cdot {{\mathbb{I}}}_{B}\left({{{{{\boldsymbol{X}}}}}}\right)\right] ={{\mathbb{E}}}_{P} \left[{{{{{{\rm{Y}}}}}}}_{{{{{{\rm{k}}}}}}}^{2}\cdot {{\mathbb{I}}}_{B}\left({{{{{\boldsymbol{X}}}}}}\right)\right]-{{\mathbb{E}}}_{P}^{2}\left({Y}_{k}\right)\le {{\mathbb{E}}}_{P}\left[{{{{{{\rm{Y}}}}}}}_{{{{{{\rm{k}}}}}}}^{2}\cdot {{\mathbb{I}}}_{B}\left({{{{{\boldsymbol{X}}}}}}\right)\right]+{{\mathbb{E}}}_{P}\left[{{{{{{\rm{Y}}}}}}}_{{{{{{\rm{k}}}}}}}^{2}\cdot {{\mathbb{I}}}_{A}\left({{{{{\boldsymbol{X}}}}}}\right)\right]-{{\mathbb{E}}}_{P}^{2}\left({Y}_{k}\right)={{\mathbb{E}}}_{P}\left[{Y}_{k}^{2}\right]-{{\mathbb{E}}}_{P}^{2}\left({Y}_{k}\right)={\sigma }_{P}^{2}({\widetilde{\mu }}^{(k)}).$$
  *End of proof*.
3. Proof of $${\sigma }_{P}^{2}({\widetilde{\mu }}^{\left(k\right)})\ge {10}^{r}\cdot {\sigma }_{P}^{2}({\hat{\mu }}^{(k)})$$**:**

$${\sigma }_{P}^{2}({\hat{\mu }}^{(k)})={{\mathbb{E}}}_{P}\left[{Y}_{k}^{2}\cdot {{\mathbb{I}}}_{B}\left({{{{{\boldsymbol{X}}}}}}\right)\right]-{{\mathbb{E}}}_{P}^{2}\left({Y}_{k}\right)={{\mathbb{E}}}_{P}\left({Y}_{k}^{2}\right)\cdot {{\mathbb{E}}}_{P}\left({{\mathbb{I}}}_{B}\left({{{{{\boldsymbol{X}}}}}}\right)\right)-{{\mathbb{E}}}_{P}^{2} \left({Y}_{k}\right)\le {{\mathbb{E}}}_{P}\left({Y}_{k}^{2}\right)\cdot {{\mathbb{E}}}_{P}\left({{\mathbb{I}}}_{B}\left({{{{{\boldsymbol{X}}}}}}\right)\right)-{{\mathbb{E}}}_{P}^{2}\left({Y}_{k}\right)\cdot {{\mathbb{E}}}_{P}\left({{\mathbb{I}}}_{B}\left({{{{{\boldsymbol{X}}}}}}\right)\right)={{\mathbb{E}}}_{P}\left({{\mathbb{I}}}_{B}\left({{{{{\boldsymbol{X}}}}}}\right)\right)\cdot \left[{{\mathbb{E}}}_{P}\left({Y}_{k}^{2}\right)-{{\mathbb{E}}}_{P}^{2}\left({Y}_{k}\right)\right]={10}^{-r}\cdot {\sigma }_{P}^{2}({\widetilde{\mu }}^{(k)}).$$

*End of proof*.
